# Integrative redescription of *Sprostoniella micrancyra* Cezar, Luque and Amato, 1999 (Monopisthocotylea: Capsalidae) and the phylogenetic position of *Sprostoniella* Bychowsky and Nagibina, 1967

**DOI:** 10.1007/s11686-026-01289-y

**Published:** 2026-05-25

**Authors:** Arthur Bessi Machado, Raquel de Oliveira Simões, Jhon Darly Chero, Arnaldo Maldonado Junior, Marcos Antonio José dos Santos, José Luis Luque

**Affiliations:** 1 Programa de Pós-Graduação em Biologia Animal, Universidade Federal Rural do Rio de Janeiro, Seropédica, Brazil; 2 Departamento de Parasitologia Animal, Universidade Federal Rural do Rio de Janeiro, Seropédica, Brazil; 3 Laboratorio de Zoología de Invertebrados, Universidad Nacional Mayor de San Marcos, Lima, Peru; 4 Laboratório de Biologia e Parasitologia de Mamíferos Silvestres Reservatórios, Fundação Instituto Oswaldo Cruz, Rio de Janeiro, Brazil; 5 Departamento de Biologia Animal, Universidade Federal Rural do Rio de Janeiro, Seropédica, Brazil

**Keywords:** Atlantic spadefish, Gill parasites, Ultrastructure, Molecular markers, Phylogenetic analysis, Capsalidae

## Abstract

**Purpose:**

The present study provides an integrative redescription of *Sprostoniella micrancyra* Cezar, Luque and Amato, 1999 (Monopisthocotylea: Capsalidae), a parasite of the Atlantic spadefish, *Chaetodipterus faber* (Broussonet, 1782) (Actinopterygii: Ephippidae) off the southeastern Brazilian coast. An additional specimen of *Sprostoniella lamothei* Pérez-Ponce de Léon and Mendoza-Garfias, 2000 collected from *Parapsettus panamensis* (Steindachner, 1876) in Peru was included for comparative molecular assessments.

**Methods:**

*S. micrancyra* specimens were examined using light microscopy, histology, and scanning electron microscopy, with morphological preparations carried out following standard processing protocols. Genomic DNA was extracted from both *Sprostoniella* species, and partial sequences of the 28S rDNA and mitochondrial *cox*1 genes were generated. Phylogenetic relationships were inferred using Maximum Likelihood and Bayesian inference.

**Results:**

Morphological observations of *S*. *micrancyra* revealed several previously unreported features, including the presence of a pair of accessory haptoral sclerites, a pair of anchors, a haptor with one central loculus, and the absence of a vaginal opening, contrasting with the original description. Scanning electron microscopy confirmed haptoral organization and detailed the structure of adhesive discs and peduncle musculature, while histology elucidated the organization of reproductive and attachment structures and muscular systems. Phylogenetic analyses of ribosomal and mitochondrial markers recovered the two *Sprostoniella* species as a monophyletic clade.

**Conclusion:**

Our results expand current knowledge of *S. micrancyra* and underscore the importance of integrative approaches to clarify the taxonomy and evolutionary relationships of capsalids.

**Supplementary Information:**

The online version contains supplementary material available at 10.1007/s11686-026-01289-y.

## Introduction

The genus *Sprostoniella* Bychowsky and Nagibina, 1967 (Capsalidae) currently comprises three recognized species, namely, its type species *Sprostoniella multitestis* Bychowsky and Nagibina, 1967; *Sprostoniella micrancyra* Cezar, Luque and Amato, 1999; and *Sprostoniella lamothei* Pérez-Ponce de León and Mendoza-Garfias, 2000 [1, 2]. These capsalids parasitize ephippid fishes of the genera *Chaetodipterus* Lacepède, 1802, *Platax* Cuvier, 1816 and *Parapsettus* Steindachner, 1876 in both Atlantic and Indo-Pacific oceans [2–8]. The genus is primarily characterized by a haptor with 17 peripheral loculi, a pair of accessory sclerites, a pair of submarginal anchors, two juxtaposed fields of multiple testes, and absence of a vagina [2, 3].

The species *S*. *micrancyra* was originally described from the Atlantic spadefish, *Chaetodipterus faber* (Broussonet, 1782) (Actinopterygii: Ephippidae), collected off the southeastern coast of Brazil [5]. This host, the only Atlantic representative of the family Ephippidae, is widely distributed from southern Brazil to the northeastern United States [9]. This parasitic species is characterized by the arrangement of septa and loculi in haptor, and relative size of the first anchor pair [5]. To date, *C. faber* remains the only known host for this parasite.

Capsalids are of particular interest because several species are important pathogens of fish, especially in aquaculture [10]. Nevertheless, detailed histological studies of capsalids are scarce and have typically focused on host pathology rather than parasite anatomy [11–14].

In this study, *S. micrancyra* is redescribed based on newly collected specimens of *C. faber* collected off the coast of Rio de Janeiro, southeastern Brazil, using a combination of morphological, SEM, histological and molecular analyses. Furthermore, we investigate the phylogenetic position of *Sprostoniella* spp. within the Capsalidae based on analyses of partial 28S rDNA and *cox*1 sequences.

## Materials and methods

### Host and parasite collection

Twenty specimens of *C. faber* and one specimen of the Panama spadefish, *Parapsettus panamensis* (Steindachner, 1876) (Actinopterygii: Ephippidae) were obtained from artisanal fishermen at local markets, respectively, in Rio de Janeiro State, southeastern Brazil, during July and August 2024, and in Lambayeque, northern Peruvian coast, during September 2025. Fish were identified according to Menezes and Figueiredo [9] and Chirichigno and Vélez [15], respectively. Gills were excised, rinsed over a 150 µm mesh sieve, and examined under a stereomicroscope for parasite detection. Parasites were detached from the gill using a fine brush and small-gauge needles.

### Morphological preparation

Parasite specimens were preserved in 4% formalin, Bouin’s fluid, or absolute ethanol depending on subsequent analyses. Specimens of *S*. *micrancyra* (n = 24) were stained with hydrochloric carmine [16], cleared with clove oil, and mounted in Canada balsam. One specimen of *S. micrancyra* was prepared with Hoyer’s medium to enhance visualization of sclerotized structures. In *P*. *panamensis*, only a single specimen of *S*. *lamothei* was found. This individual was divided, with the haptoral region mounted in Hoyer’s medium for morphological analysis of sclerotized structures, while the remaining portion was preserved in absolute ethanol for molecular analyses.

### Scanning electron microscopy (SEM)

Four specimens of *S. micrancyra* were dehydrated through an ethanol series, critical-point dried in CO₂ with a Tousimis Autosamdri-815 Critical Point Dryers, sputter-coated with gold with a Cressington 108 Auto Sputter Coater, and examined with a JEOL JSM-6390 scanning electron microscope at Rudolf Barth Electron Microscopy Platform, Oswaldo Cruz Institute (IOC), Rio de Janeiro, Brazil.

### Histology

Ten specimens of *S. micrancyra* fixed in Bouin’s fluid were transferred to 70% ethanol, embedded in paraffin, and sectioned at 5 µm thickness with a semi-automatic RMC rotary microtome. Slides were stained with hematoxylin–eosin (HE) and Gomori’s trichrome (GT) following Abrahamsohn [17]. On average, six histological sections per specimen were analyzed.

### Microscopy and illustrations

Slides were examined and photographed under an Olympus BX51 microscope using Capture 2.4 software. Line drawings were prepared in Inkscape. Measurements are in micrometers, with ranges followed by means in parentheses. Voucher specimens of *S. micrancyra* (paragenophores, in whole-mounted and histological slides) and of *S. lamothei* (hologenophore, in Hoyer’s medium slide) were deposited in the Helminthological Collection of the Oswaldo Cruz Institute (CHIOC), Rio de Janeiro (Brazil), and in the Helminthological Collection of the Museum of Natural History at the San Marcos University (MUSM-HEL), Lima (Peru).

### DNA extraction and PCR amplification

Genomic DNA was extracted from a single specimen of both *Sprostoniella* species using the QIAamp DNA Mini Kit (Qiagen). The partial 28S rDNA was amplified by polymerase chain reaction (PCR) using the primers C1/D2 [18]. The mitochondrial DNA *cox*1 partial fragment was amplified using the primers COI-ASmit1/COI-ASmit2 [19]. PCR reactions were performed with Promega PCR Master Mix under conditions described by Ogawa et al. [20] for 28S rDNA and Verneau et al. [21] for *cox*1. Amplicons were purified using QIAquick PCR Purification Kit (Qiagen).

### Sequencing and phylogenetic analysis

Sequencing was performed with BigDye Terminator v3.1 on ABI 3730 DNA Analyzer at RPT01A Sequencing Platform (IOC, Rio de Janeiro). Consensus sequences were assembled in Geneious R9.1 [22] and deposited in GenBank under accession numbers PZ328782 (*S. micrancyra* 28S rDNA), PZ323883 (*S. lamothei* 28S rDNA), PZ284734 (*S. micrancyra cox*1) and PZ284680 (*S. lamothei cox*1).

Alignments were performed separately for 28S rDNA and *cox*1. Best-fit nucleotide substitution models were selected using the “Best DNA Model” finder in MEGA 11 [23]. Maximum Likelihood (ML) and Bayesian inference (BI) analyses were run in MEGA 11 [23] and MrBayes 3.2.6 [24] through CIPRES Science Gateway [25], respectively. Nodal support was evaluated with 1,000 bootstrap replicates (ML) and posterior probabilities (BI). In both analyses, sequences from representatives of Capsalidae were included. Microbothriidae and Monocotylidae sequences were used as outgroups in the 28S rDNA analysis, whereas a Monocotylidae sequence was used as the outgroup for the *cox*1 analysis. The Supplementary Information file lists the GenBank reference sequences used in the phylogenetic analyses of 28S rDNA and *cox*1.

## Results

*Sprostoniella micrancyra* from *C. faber* was molecularly characterized and is redescribed below. A single specimen of *S. lamothei* from *P. panamensis* was used for sequencing and the hologenophore was deposited in CHIOC, Rio de Janeiro (Brazil), under the deposit number 41037.

Family Capsalidae Baird, 1853.

Genus *Sprostoniella* Bychowsky and Nagibina, 1967.

*Sprostoniella micrancyra* Cezar, Luque and Amato, 1999.

### Redescription

**Fig. 1:**
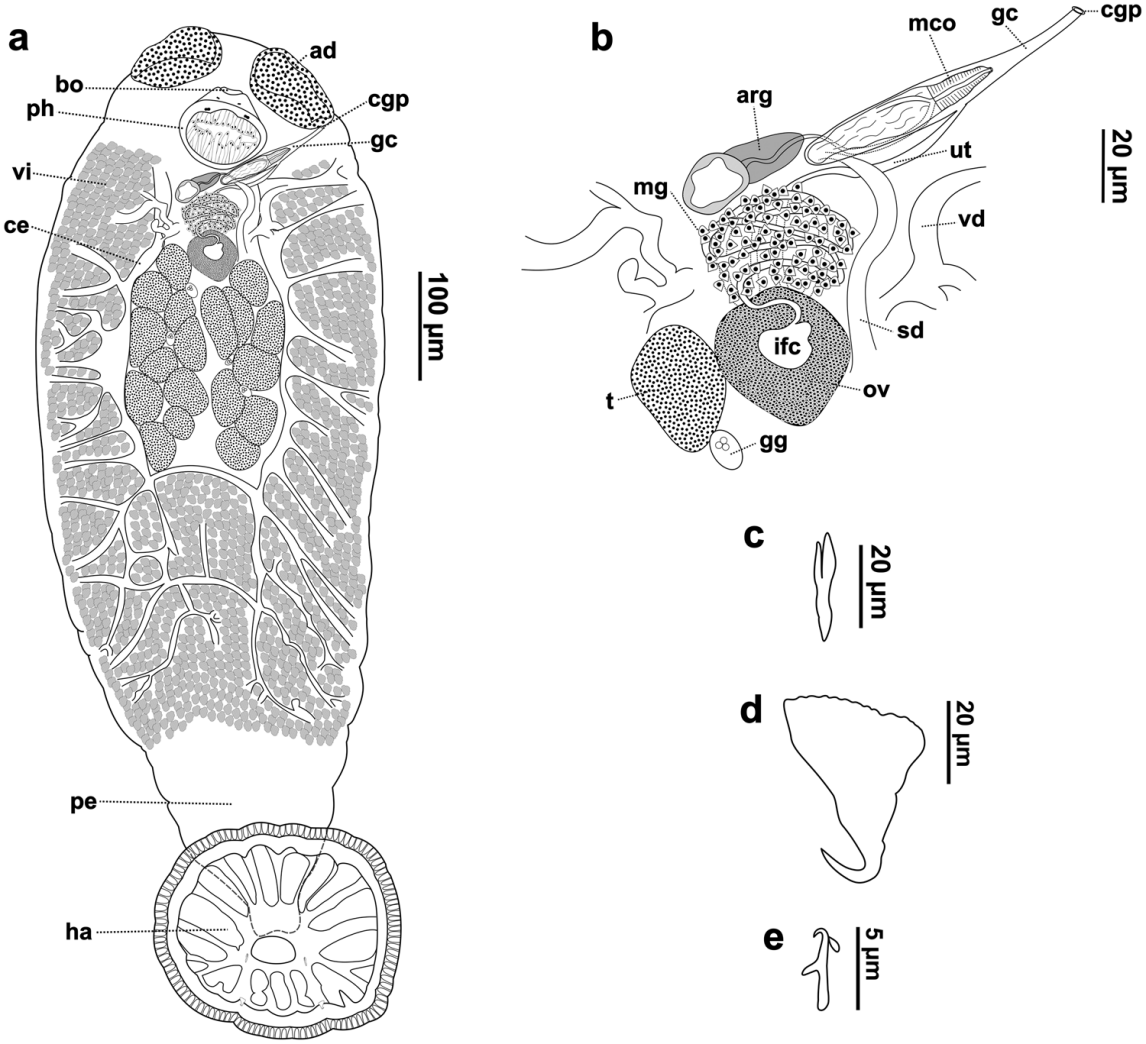
*Sprostoniella micrancyra* ex *C. faber*. **a** Entire body, ventral view, **b** reproductive system, **c** accessory sclerite, **d** second anchor, **e** hook. Abbreviations: ad adhesive disc; bo buccal organ; ph pharynx; vi vitellaria; cgp common genital pore; gc genital canal; ce ceca; pe peduncle; ha haptor; mco male copulatory organ; arg accessory reserve gland; ov ovary; ifc internal fertilization chamber; ut uterus; mg Mehlis’ glands; vd vitellinic duct; sd spermatic duct; t testicle; gg Goto’s gland

**Fig. 2:**
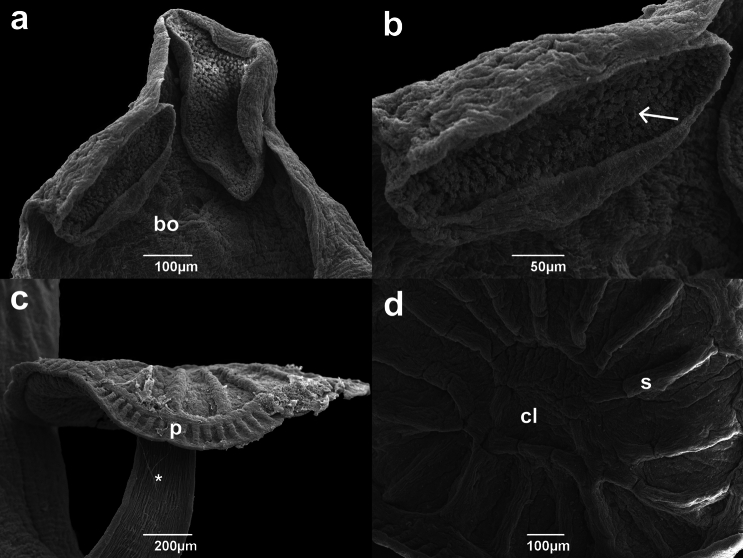
Scanning electron micrographs of *S. micrancyra* ex *C. faber*. **a** Anterior region showing adhesive discs and buccal organ, **b** detail of adhesive disc surface with secretory projections, **c** haptor in ventral view, **d** detail of haptoral septa. Abbreviations: bo buccal organ; p papillary sheath; cl central loculus; s septa; * strong cords of muscle fibers; arrow indicates secretory lumps

**Fig. 3:**
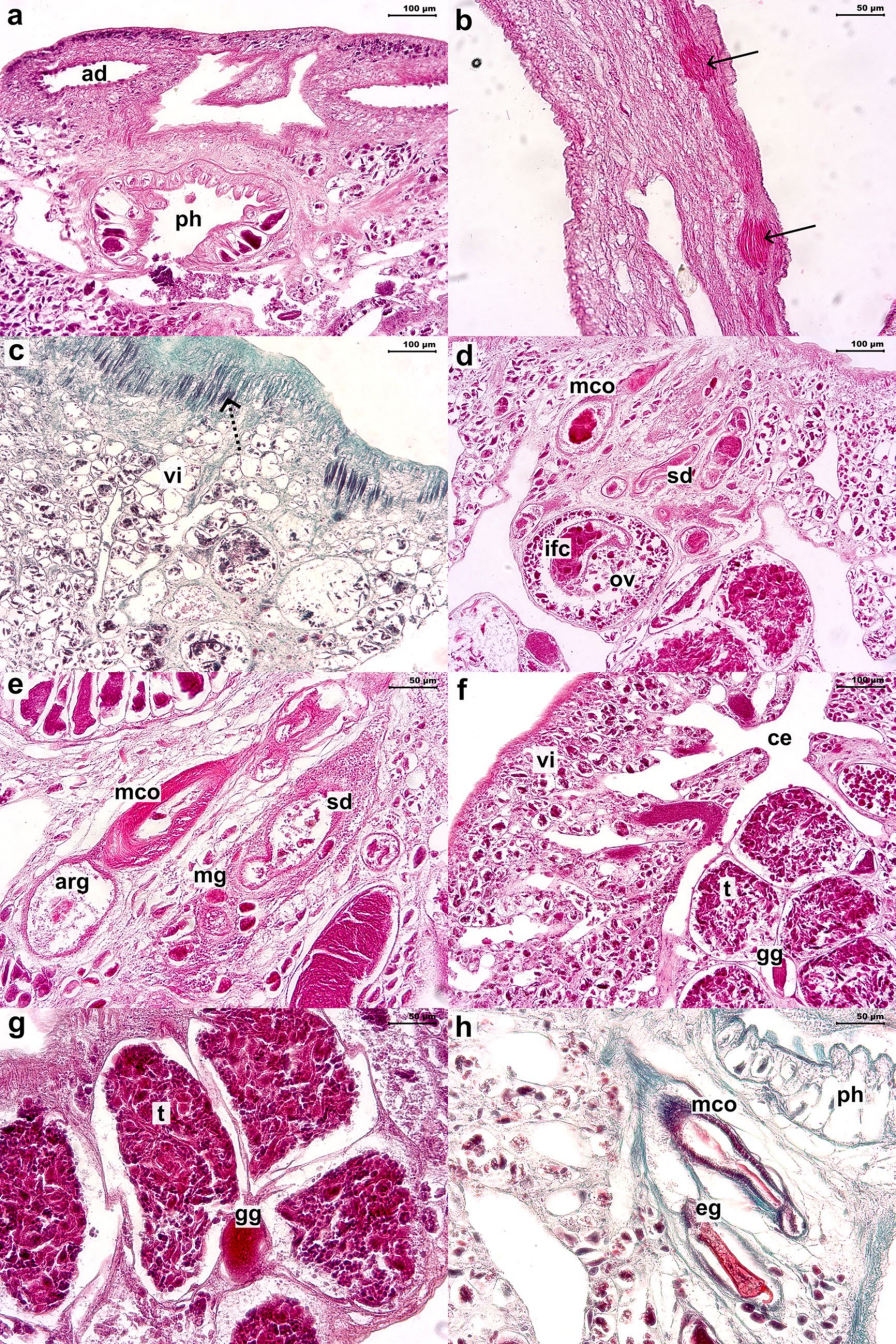
Histological sections of *S. micrancyra* ex *C. faber* in longitudinal sections, stained in HE (**a**, **b**, **d**–**g**) and GT (**c** and **h**). **a** Anterior extremity, **b** peduncle musculature, **c** subtegumentary muscle bundles beneath adhesive discs, **d** reproductive structures, **e** mco, in detail, **f** cecal branches, testicular units and Goto’s glands, **g** testicular follicles and Goto’s glands, in detail, **h** MCO and egg. Abbreviations: ad adhesive disc; ph pharynx; vi vitellaria; mg Mehlis’ glands; mco male copulatory organ; arg accessory reserve gland; sd spermatic duct; ov ovary; ifc internal fertilization chamber; t testicle; ce cecal branch; gg Goto’s gland; eg egg; arrow indicates dilated bulb-shaped regions along the muscle cord axis; dotted arrow indicates muscle bundles network in subcutaneous region

Description based on 24 specimens. Measurements in Table 1. Body flattened, elliptical, larger than wider, greatest width at ovary level (Fig. 1a). Smooth tegument, covered by squamous to cubic epithelium; subcutaneous region composed of network of small muscle bundles arranged in parallel and perpendicular directions, in lamina propria (Fig. 2; 3c). Haptor round, pedunculated, with peripheral papillary sheath; lined by squamous epithelium; with one central loculus; 17 muscular septa, two bifid and two incomplete (Fig. 1a; 2c, d; 3b). Accessory haptoral sclerite arrow-shaped, with bifid base (Fig. 1c). Anterior anchors absent, posterior anchors flattened bilaterally, each with irregular serrated base, short shaft, recurved point (Fig. 1d). Fourteen marginal hooks located along haptor periphery, near transition with papillary sheath; each comprised of arcing shaft and short point, protruding thumb, shank comprising single subunit (Fig. 1e). Peduncle long and muscular, especially in posterior portion; longitudinal cords of muscle bundles with dilated bulb-shaped regions along axis; inserted in haptor in central region (Fig. 1a; 2a; 3b). Two adhesive discs at anterior end, ventral surface; composed of lumen and secretory epithelium; externally organized by units of secretory lumps; cells with acidophilic cytoplasm and cytoplasmic granules, columnar to domed shaped; vacuolated cells in lamina propria (Fig. 1a; 2a, b; 3a). Four eyes organized in two rows pairs, between adhesive structures and buccal organ (Fig. 1a). Buccal organ medially, at adhesive discs posterior portion level, anterior to pharynx (Fig. 1a; 2a). Pharynx surrounded by thin capsule of connective tissue; composed of papillary projections; shorter upper projections formed by projections of lining epithelium and lamina propria; longer lower projections formed by acidophilic and granular content, with thin capsule of connective tissue (Fig. 1a; 3a). Intestinal ceca highly branched in anterior and middle body, reaching subcutaneous region; lined by squamous epithelium (Fig. 1a; 3f). Two testicular fields with 7–10 testes each, composed of testicles units; each testicle is composed of different cells of germ line, flattened cells in periphery and cells resting on basal lamina, and surrounded by thin connective tissue (Fig. 1a, b; 3f, g). Four Goto’s glands, between testicles; rounded, acidophilic with cytoplasmic granules, lined by low cuboidal epithelium (Fig. 1a, b; 3f, g). Spermatic ducts lined by squamous to cuboidal epithelium (Fig. 3e, f). Male copulatory organ (MCO) muscular, sinistrolateral to pharynx; anterior portion surrounded by thin muscular capsule, with internal duct lined by columnar to squamous epithelium (Fig. 1a, b; 3d, e, h). Accessory reserve gland posterior to MCO, composed of two chambers; anterior chamber with muscular fibers organized concentrically around internal duct; posterior chamber lined by thin epithelium layer, with internal content (Fig. 1a, b; 3e). Accessory reserve gland duct joining spermatic duct approximately at MCO mid-region (Fig. 1a, b). Genital canal enclosing MCO (Fig. 1a, b). Common genital pore located on left side, near posterolateral end of adhesive gland (Fig. 1a). Ovary surrounded by thin connective tissue capsule (Fig. 3d). Internal fertilization chamber (IFC) within ovary, lined by squamous epithelium and thin lamina propria (Fig. 1a, b; 3d). Uterus convoluted, anterior to ovary, partially overlapping the posterior region of MCO and accessory reserve gland. (Fig. 1a, b). Mehlis’ glands numerous, surrounding and partially overlapping uterus; piriform, acidophilic with cytoplasmic granules (Fig. 1a, b; 3e). Terminal portion of uterus joining genital canal in medial region (Fig. 1a, b). Vitellarium dense, along almost entire length of body, with exception of peduncle and haptor; organized in form of clump and composed of acidophilic structures with granules, in spaces surrounded by thin membrane of connective tissue (Fig. 1a; 3c, f). Vitellarium reservoir not observed. Egg sclerotized, with filamentous appendage, of acidophilic nature (Fig. 3h).

**Table 1:** Comparative morphometric data (µm) of *Sprostoniella micrancyra* and congeners, based on original descriptions and redescriptions, range (minimum–maximum) followed by the mean in parentheses

Feature					
Species	*Sprostoniella micrancyra* Cezar, Luque and Amato, 1999	*Sprostoniella micrancyra* Cezar, Luque and Amato, 1999	*Sprostoniella multitestis* Bychowsky and Nagibina, 1967	*Sprostoniella* cf. *multitestis* Bychowsky and Nagibina, 1967	*Sprostoniella lamothei* Pérez-Ponce de Léon and Mendoza-Garfias, 2000
Host species	*Chaetodipterus faber* (Broussonet, 1782)	*Chaetodipterus faber* (Broussonet, 1782)	*Platax pinnatus* (Linnaeus, 1758)	*Platax teira* (Fabricius, 1775)	*Chaetodipterus zonatus* (Girard, 1858)
Locality	Brazilian Southeast	Brazilian Southeast	South China Sea	Queensland, Australia	Mexican Western coast
Body length	1903–9474 (6150) (n = 24)	3200–8300 (6300)	3000–3400	2120–4090 (2910)	2175–5175 (3957)
Body width	683–2778 (1890) (n = 24)	1100–2700 (2200)	1600–2000	1410–2960 (2360)	110–2850 (1939)
Anterior attachment organ length	197–794 (416) (n = 48*)	NM	530	524–816 (647)	250–462 (338)
Anterior attachment organ width	272–805 (547) (n = 48*)			412–639 (508)	337–575 (388)
Haptor length	525–2969 (1615) (n = 24)	1300–2200 (1600)	900–950	1080–1750 (1390)	537–1675 (1005)
Haptor width	558–2615 (1719) (n = 24)			1070–1810 (1510)	775–1737 (1391)
Accessory Sclerite length^a^	18–38 (26) (n = 24)	31	170–220	102–208 (157)^b^	30–45 (39)^c^
Second anchor length	20–48 (37) (n = 24)	27	36	35–43 (37)	30–42 (34)^d^
Hook length	5–8 (6) (n = 10)	6	12	13–14	6–8 (7)
Buccal organ length	45–139 (73) (n = 24)	274–640 (426)	NM	NM	NM
Buccal organ width	64–868 (517) (n = 24)	366–421 (522)			
Pharynx length	114–415 (317) (n = 24)	219–540 (500)	230–320	177–383 (284)	175–450 (321)
Pharynx width	184–588 (457) (n = 24)	275–603 (461)	420–460	363–673 (526)	212–550 (409)
Testes length	40–539 (277) (n = 24)	146–348 (256)	NA	185–416 (271)	45–153 (104)
Testes width	46–382 (237) (n = 24)		NA	NA	30–120 (79)
Testes number in left group	7–10 (8) (n = 24)	8–10	7–9	7–10 (9)	8–10
Testes number in right group	7–10 (8) (n = 24)	9–10			8–10
Goto’s gland length	75–156 (114) (n = 15)	109–146 (127)	NM	NM	60–137 (97)
Goto’s gland width	47–126 (67) (n = 15)	43.8–51 (47)			10–81 (55)
Penis pouch length	345–606 (472) (n = 15)	175–292 (235)	NM	NM	135–600 (281)
Penis pouch width	75–135 (102) (n = 15)	51–131 (84)			30–87 (58)
Ovary length	69–485 (276) (n = 16)	164–495 (365)	NM	132–274 (195)	105–387 (263)
Ovary width	50–603 (379) (n = 16)			161–392 (281)	108 – 425 (279)
Reference	Present study	Cezar et al. [5]	Bychowsky and Nagibina [3]	Kritsky [2]	Pérez-Ponce de Léon and Mendoza-Garfias [6]

^*^ Both left and right anterior attachment organs length and width were measured. NM: not measured

^a^corresponding to the “first pair of anchors” in Cezar et al. [5]

^b^referred to in Kritsky [2] as “accessory haptoral sclerite”

^c^referred to in Pérez–Ponce de Léon and Mendoza-Garfias [6] as “accessory sclerites”

^d^referred to in Pérez–Ponce de Léon and Mendoza-Garfias [6] as “posterior hamuli”

### Taxonomic summary

*Type and only host: Chaetodipterus faber* (Broussonet, 1782).

*Type and only locality:* coastal zone of the State of Rio de Janeiro, southeastern Brazil.

*Site of infection:* Gill filaments.

*Infestation parameters:* Total number of hosts: 20, prevalence: 85%, total number of parasites: 73, mean intensity: 4.3, mean abundance: 3.6, range of intensity: 1 – 17.

*Deposited material:* Vouchers CHIOC no 41036a-c (whole mounted slides) and 41036d (histological slides), and MUSM-HEL no 5640 (whole mounted slide).

*Examined material:* Holotype CHIOC no 34000a, paratypes (three whole-mounted) CHIOC no 34000b-d.

### Molecular and phylogenetic analysis

**Fig. 4:**
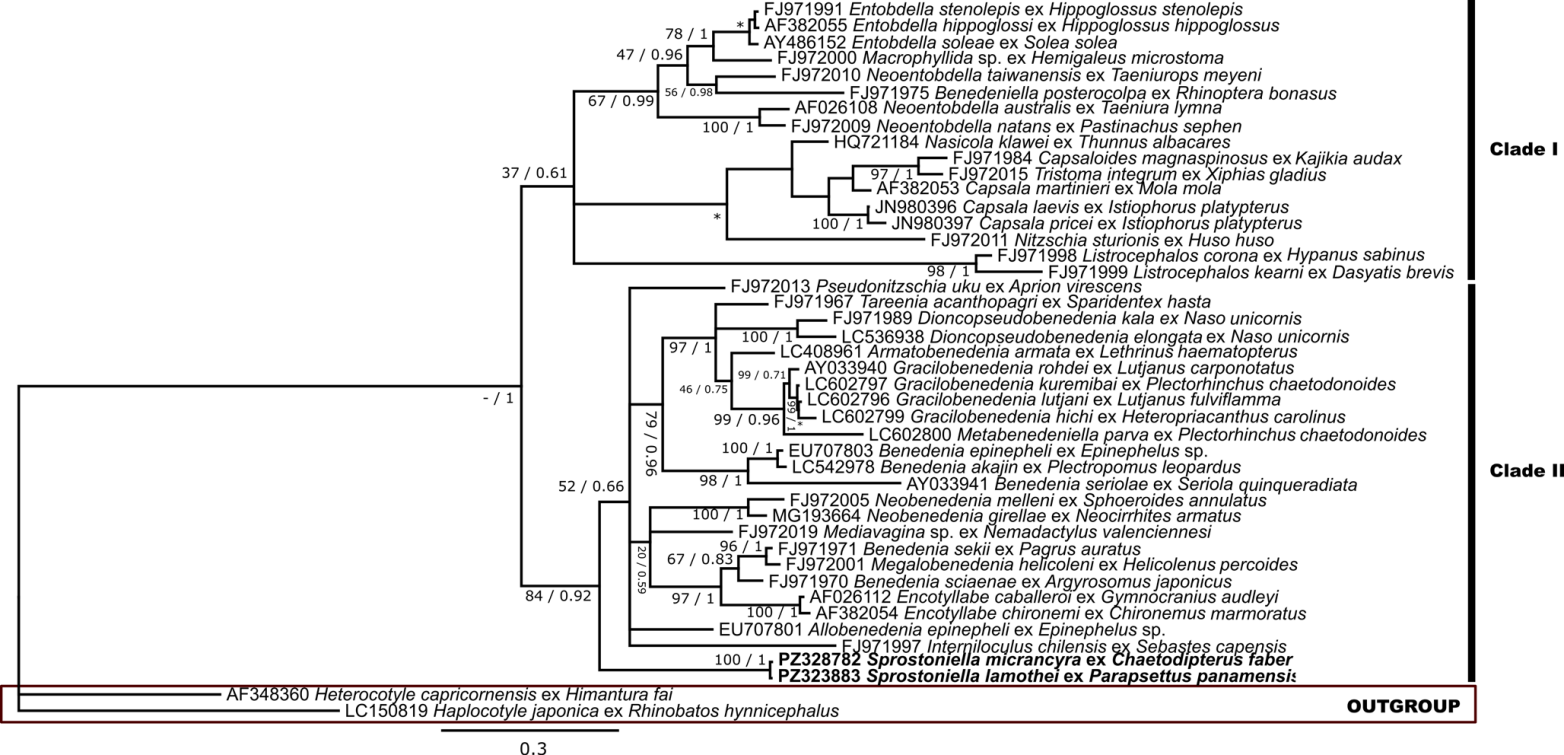
Phylogenetic relationships among 28S rDNA sequences of Capsalidae, including *S. micrancyra* ex *C. faber* and *S. lamothei* ex *P*. *panamensis* (in bold). The tree was inferred by using the ML and BI. Evolutive model used was General Time Reversible, with gamma distributed with invariant sites (GTR + G + I). The nodal support is described at the left by bootstrap replicates and at the right by posterior probability. Scale bar indicates number of substitutions per site.—indicates that this node value was not evaluated and * indicates a distinct clade topology between ML and BI analyses

28S rDNA alignment consisted of 44 taxa and 894 characters, of which 258 were conserved, 621 were variable, and 566 were parsimony-informative. Bayesian inference (BI) returned a mean estimated marginal likelihood of -12,384.237, with a median value of -12,383.9. Effective sample size (ESS) for all parameters was 893.417. Maximum Likelihood (ML) and BI analyses yielded similar topologies, showing only minor differences in clade arrangement. Final topology presented (Fig. 4) is based on BI tree.

**Fig. 5:**
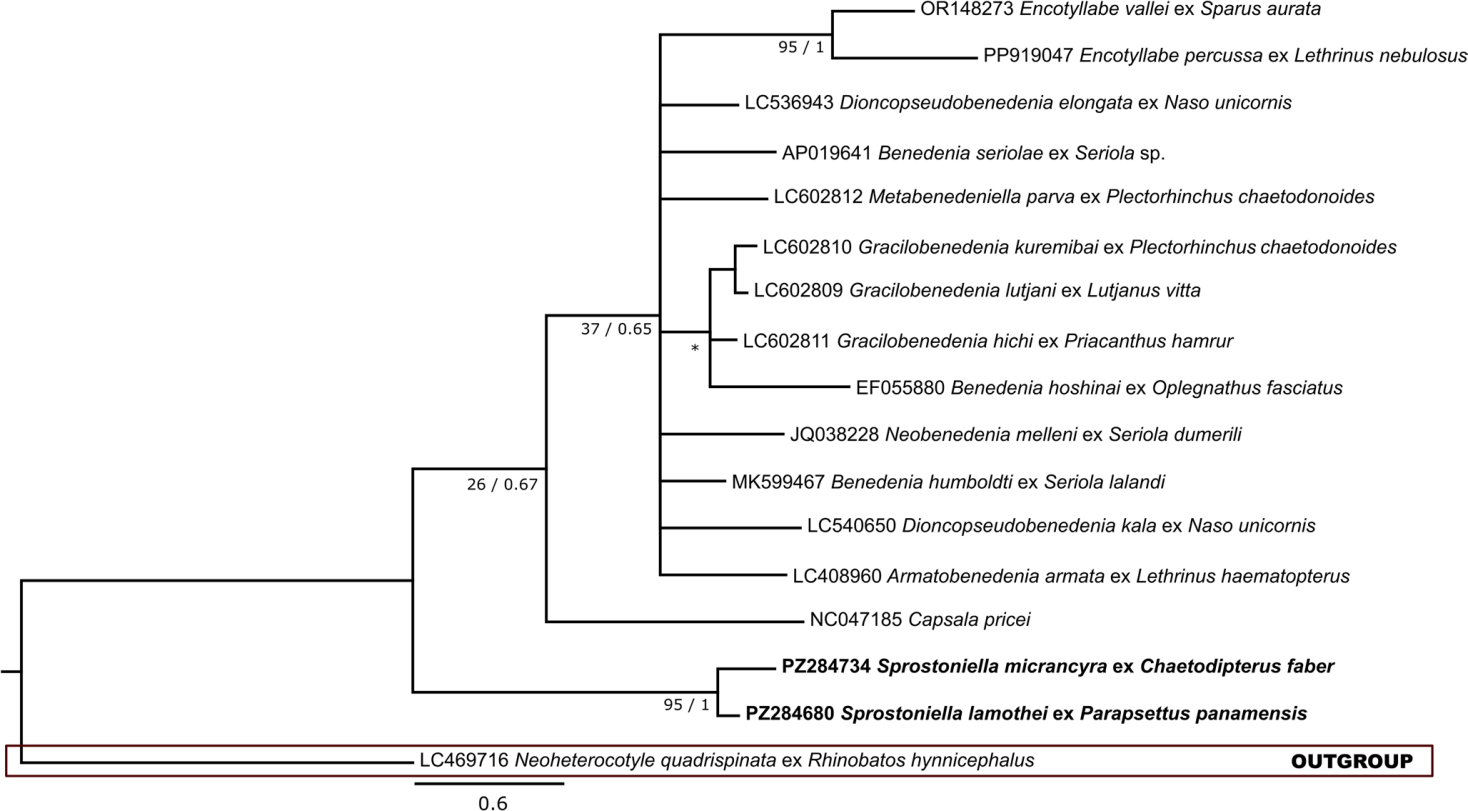
Phylogenetic relationships among *cox*1 sequences of Capsalidae, including *S. micrancyra* ex *C. faber* and *S. lamothei* ex *P*. *panamensis* (in bold). The tree was inferred by using the ML and BI. Evolutive model used was General Time Reversible, with gamma distributed with invariant sites (GTR + G + I). The nodal support is described at the left by bootstrap replicates and at the right by posterior probability. Scale bar indicates number of substitutions per site. * indicates a distinct clade topology between ML and BI analyses

*Cox*1 alignment included 17 taxa and 422 characters, of which 243 were conserved, 179 were variable, and 145 were parsimony-informative. Bayesian analysis returned a mean estimated marginal likelihood of -2860.067, with a median value of -2859.785. ESS for all parameters was 1788.665. ML and BI analyses yielded similar topologies, showing only minor differences in clade arrangement. Final topology for mitochondrial marker was likewise inferred from BI analysis (Fig. 5).

In both ribosomal and mitochondrial phylogenetic analyses, the sequences of *S. micrancyra* and *S. lamothei* clustered together with high support values in both ML (100% and 95%, respectively) and BI (1 in both) topologies. In topology derived from ribosomal analysis, inclusion of *Sprostoniella* sequences resulted in the family Capsalidae being arranged into two major clades.

### Taxonomic remarks and comparative morphology

Overall, morphometric data of *S*. *micrancyra* specimens analyzed in this study differed from those reported by Cezar et al. [5] (Table 1). Moreover, in our specimens both testicular fields contained 7–10 testes each, whereas the original description of *S*. *micrancyra* reported a different number, 8–10 in the left field and 9–10 in the right.

Cezar et al. [5] described three pairs of anchors in *S. micrancyra*. However, in the present material, only one pair of anchors and one pair of accessory haptoral sclerites were observed, corresponding to the second and third pairs of the original description. Additionally, the position of the accessory haptoral sclerite varied within the haptor and occupied the region described by Cezar et al. [5] as belonging to both the first and third pairs. This suggests that the previously reported first anchor pair may not exist and was likely a positional artifact of the third pair, which in fact represents the accessory haptoral sclerite. Moreover, the shape of the first anchor pair illustrated in the original description differs markedly from that observed in the present study, the latter being more consistent with the anchor morphology typical of its congeners [2, 3, 6].

According to Bychowsky and Nagibina [3], presence of one pair of accessory sclerites and one pair of anchors are key taxonomic features of the genus. This pattern, typically accompanied by 14 hooks, is consistently observed across multiple species, supporting its value as a defining characteristic. For instance, *S. multitestis* and *S. lamothei* both exhibit two anchor pairs and 14 hooks [2, 3, 6].

Cezar et al. [5] reported the presence of a vaginal opening in *S. micrancyra*, however, this structure was not illustrated. In contrast, the present morphological and SEM analyses revealed no evidence of such a structure. The absence of a vagina has also been reported in *S. lamothei* [6]. In the original description of *S. multitestis*, both a common genital atrium and a vaginal opening were described [3]. Nevertheless, Kritsky [2], in his description of *Sprostoniella* cf. *multitestis*, emphasized the absence of a vagina in the examined specimens.

Furthermore, in the specimens of *S. micrancyra* analyzed in this study, only a single central loculus was observed in the haptor, which contradicts the original description by Cezar et al. [5]. In the amended diagnosis of *Sprostoniella*, Kritsky [2] stated that members of the genus possess a single central loculus surrounded by 17 peripheral loculi. However, the original description of *S*. *micrancyra* reported two central loculi, a feature that was even proposed as a distinguishing characteristic separating this species from its congeners. Examination of our specimens revealed that only one true central loculus is present, and that the supposed second central loculus is an artifact, most likely a raised area produced by the dorsal insertion of the peduncle into haptor.

Bychowsky and Nagibina [3] hypothesized that, in *Sprostoniella*, two ancestral testicles may have undergone subdivision into multiple follicular units over the course of evolution. The present histological findings support this hypothesis, demonstrating two lateral clusters of testicular fields composed of testicular units, with a connective tissue around it. The physical delimitation and separation of each unit by a distinct connective tissue layer support interpretation that each unit represents an individual testicle. This observation is consistent with previous descriptions of congeners, where the terminology “multiple testes” is employed, or alternatively, each unit is interpreted as an individual testicle [2, 5, 6]. Additionally, a single egg was observed near the terminal region of the uterus. Acidophilic in nature, the egg possesses a thick shell and a partially visible filamentous appendage, characteristic of the group, used for attachment to the host [26].

*Sprostoniella micrancyra* differs from its congeners by possessing a MCO positioned sinistro-laterally to pharynx, whereas remaining species exhibit a copulatory organ situated closer to posterior pharyngeal margin. Although Bychowsky and Nagibina [3] illustrated the MCO of *S. multitestis* in a position more similar to that observed in *S. micrancyra*, Kritsky [2] emphasized its more posterior placement relative to pharynx. In addition, *S*. *micrancyra* can also be distinguished from *S. multitestis* by presence of two bifurcated and two incomplete septa, a longer peduncle, and a shorter first anchor pair. *Sprostoniella micrancyra* differs from *S. lamothei* by possessing larger reproductive structures, a longer peduncle, and intestinal ceca that do not extend toward the haptor.

Identification Key for Species of *Sprostoniella*

1a. Testes smaller than ovary . . . . . . . . . . . . . . . . . . . . . . . . . . . . . . . . . . . . . . . . . . . . . . . . . . . . . *Sprostoniella lamothei*

1b. Testes overlapping the size of the ovary . . . . . . . . . . . . . . . . . . . . . . . . . . . . . . . . . . . . . . . . . . . . . . . . . . . . . . . . 2

2a. MCO sinistrolateral to pharynx, first pair of anchors short (18–38 µm) . . . . . . . . . . . . . . . . . . . . . . . . . . . . . . . . . . . . . . . .. . . . . . . . . . . . . . . . . . . . . . *Sprostoniella micrancyra*

2b. MCO posterior to pharynx, first pair of anchors long (102–220 µm) . . . . . . . . . . . . . . . . . . . . . . . . . . . . . . . . . . . . . . . . . . . . . . . . . . . . . . . . . . . . . . . *Sprostoniella multitestis*

## Discussion

*Sprostoniella* was originally proposed to accommodate *S. multitestis*, described from *Platax pinnatus* (Linnaeus, 1758) in the South China Sea [3]. Additional geographical and host records include *Platax orbicularis* (Forsskål, 1775) also from the South China Sea (Mamaev, 1970) [4], as well as *Sprostoniella* cf. *multitestis* parasitizing *Platax teira* (Fabricius, 1775) and *P. orbicularis* in Australia [2]. Although resembling members of *Sprostonia* Bychowsky, 1967, the species was placed in a separate genus due to its more complex haptor and distinct testicular arrangement [3]. Since then, two additional species have been described, *S. micrancyra* from *Chaetodipterus faber* off Brazil, and *S. lamothei* from *C. zonatus* off Mexico and from *P*. *panamensis* off Mexico and Peru [5–8]. Interestingly, the two Atlantic species are associated with *Chaetodipterus*, whereas the Indo-Pacific species are associated with *Platax* and *Parapsettus*. This observation may indicate a host-biogeographical pattern. However, more comprehensive studies, particularly those incorporating molecular data (both hosts and parasites), are required to substantiate this hypothesis. A fourth species, *Sprostoniella teria* Bannai and Muhammad, 2014, is listed in the World Register of Marine Species (WoRMS). However, it is considered invalid because its status does not comply with the provisions of the International Code of Zoological Nomenclature (ICZN), articles 8.5 and 16.4. This is due to the species having been published twice in different journals and to the absence of an explicit designation of type material. [1, 2].

In comparing our specimens with the original description of *S. micrancyra* [5], general morphology was consistent, but significant discrepancies emerged. The present study consistently observed only one pair of anchors (second pair), rather than three as previously reported, a single central loculus in haptor, but originally described with two loculi of unequal size, and lacking vaginal opening. These findings align the species more closely with the generic diagnosis, i.e., one accessory sclerite pair, one anchor pair, a single central loculus in haptor and absence of vagina [2, 3].

This is the first investigation of *S. micrancyra* using SEM and histology, and the results add valuable information to discussions of capsalid morphological evolution. Addressing this gap is essential for clarifying its diagnosis and refining the taxonomy of both species and genus. SEM allowed detailed visualization of tegumental surfaces, haptoral organization, and surface pores.

Histology provided novel insights into internal anatomy of *S. micrancyra*. Arrangement of several testicular units, each distinctly bounded by connective tissue, provides morphological support for the hypothesis of testicular subdivision proposed by Bychowsky and Nagibina [3] in *Sprostoniella*. Whittington [27] and Ergova [28] also characterized the genus as having multiple testicles arranged in two distinct groups. Regarding Goto’s glands, although their function is not clearly defined in literature, histological findings presented here indicate that they are secretory structures containing acidophilic cytoplasmic granules. This secretion may play a role in regulating reproductive structures, particularly the testicles due to their proximity, either by modulating their activity or by participating in post-spermatogenic processes. Muscular capsules surrounding MCO were also documented in present study.

Comparable adaptations have been reported in other parasitic species. For example, mucous-secreting cells in *Metapolystoma ohlerianum* Landman, Verneau, Vences and Du Preez, 2023 resemble adhesive-disc cells of *S. micrancyra*, while pharyngeal glands show structural parallels to papillary projections described here [29]. Secretory cells in the pharynx of *Entobdella soleae* (Van Beneden and Hesse, 1863) produce proteolytic enzymes used for epithelial feeding [30]. Although digestive physiology of *S. micrancyra* remains unknown, papillary projections observed in the pharynx likely function in mechanical digestion, whereas the secretory material present appears to play an enzymatic role.

Adhesive discs of *S. micrancyra* were densely packed with large secretory cells. Externally, SEM showed lobe-like projections likely corresponding to glandular openings. These findings are consistent with reports of attachment adaptations in polystomatids, where suckers and secretory organs provide anchorage in mechanically challenging environments [31, 32]. In *S. micrancyra*, abundant muscular bundles beneath tegument and within peduncle likely aid in resisting detachment under gill water flow, while secretory epithelia in adhesive discs may produce mucous substances for fixation.

Our molecular analyses represent the first genetic data for *S. micrancyra* and *S. lamothei*. Both ribosomal and mitochondrial analyses supported monophyly of the Capsalidae, consistent with findings of Perkins et al. [33] and Nitta [34]. The resulting topologies closely resembled those reported by Nitta [34]. However, in contrast to that study, our ribosomal analysis resolved the Capsalidae into two principal clades. The first clade comprises subfamilies Pseudonitzschiinae, Encotyllabinae, Benedeniinae 1, 2, 3 and 4, Trochopodinae 1 and 2, and Interninoculinae (including sequences analyzed by Nitta [34]), as well as others representatives of subfamily Trochopodinae, here represented by the *Sprostoniella* species sequenced in this study, but these did not cluster with the other Trochopodinae previously included by Nitta [34]. The second major clade includes the remaining representatives incorporated by Nitta [34] in his ribosomal phylogeny. In an analysis based on *cox*1 marker, support values were lower than those reported by Nitta [34]. This reduction may reflect existing gaps in molecular information for several genera that remain unsampled or insufficiently represented. When compared with 28S rDNA phylogeny presented by Perkins et al. [33], topology recovered here, now including sequences of *Sprostoniella*, shows a similar overall structure. In our analysis, the clades designated by those authors as “1a” and “1b” were recovered together within clade II, whereas their clade “2a” corresponds to clade I in the present study. Consequently, clades “1a” and “1b” formed a single lineage sharing a common ancestral branch in our reconstruction.

The presence of two testicular groups composed of multiple independent units may be a primary trait within the family, suggesting a derived evolutionary tendency toward fusion or reduction of these units in other lineages. However, this feature could also represent a homoplasy within the genus. Bychowsky and Nagibina [3], in his original description of the genus *Sprostoniella*, regarded the presence of multiple testicles as a secondary trait within the group. Therefore, future studies incorporating a broader taxonomic sampling and additional molecular markers, concomitant with morphological analyses, are recommended to achieve a clearer and more robust understanding of phylogenetic position of *Sprostoniella* and evolutionary relationships within Capsalidae.

## Supplementary Information

Below is the link to the electronic supplementary material.

Supplementary file1 (PDF 263 kb)

Supplementary file1

## Data Availability

No datasets were generated or analysed during the current study.
