# Supplementary material for: Integrative redescription of Sprostoniella micrancyra Cezar, Luque and Amato, 1999 (Monopisthocotylea: Capsalidae) and the phylogenetic position of Sprostoniella Bychowsky and Nagibina, 1967
Source: Acta Parasitol. 2026 May 25;71(3):124. doi: 10.1007/s11686-026-01289-y (PMC13201291; doi:10.1007/s11686-026-01289-y)
Supplement: Supplementary file 2 — Supplementary file1 [file 11686_2026_1289_MOESM2_ESM.pdf]

Integrative redescription of *Sprostoniella micrancyra* Cezar, Luque and Amato, 1999 (Monopisthocotylea: Capsalidae) and the phylogenetic position of *Sprostoniella* Bychowsky and Nagibina, 1967

Acta Parasitologica

E-mail address of the corresponding author: jlluque@ufrrj.br

Arthur Bessi Machado[1] (orcid: 0009-0003-8793-7380); Raquel de Oliveira Simões[2] (orcid: 0000-0001-5130-3341); Jhon Darly Chero[3] (orcid: 0000-0003-3633-1365); Arnaldo Maldonado Junior[4] (orcid: 0000-0003-4067-8660); Marcos Antonio José dos Santos[5]; José Luis Luque[2] (orcid: 0000-0003-3515-1127).

[1] Programa de Pós-Graduação em Biologia Animal, Universidade Federal Rural do Rio de Janeiro, Seropédica, Brazil.
[2] Departamento de Parasitologia Animal, Universidade Federal Rural do Rio de Janeiro, Seropédica, Brazil.
[3] Laboratorio de Zoología de Invertebrados, Universidad Nacional Mayor de San Marcos, Lima, Peru.
[4] Laboratório de Biologia e Parasitologia de Mamíferos Silvestres Reservatórios, Fundação Instituto Oswaldo Cruz, Rio de Janeiro, Brazil.
[5] Departamento de Biologia Animal, Universidade Federal Rural do Rio de Janeiro, Seropédica, Brazil.

Supplementary Table 1. Isolate sequences used in 28S rDNA molecular analyses associated with their GenBank accession numbers, host, locality and reference. * Indicates sequences used as outgroup. NA: not available.

Species	GenBank Access Number	Host	Locality	Reference
Allobenedenia epinepheli (Bychowsky and Nagibina, 1967) Yang, Kritsky and Sun, 2004	EU707801	*Epinephelus* sp.	Vietnam	NA
Armatobenedenia armata (Nitta, 2019) Nitta, 2021	LC408961	*Lethrinus haematopterus* Temminck and Schlegel, 1844	Japan	Nitta (2019) [1]
Benedenia akajin Ogawa, Mizuochi, Yamaguchi, Shirakashi, Asai and Agawa, 2021	LC542978	*Plectropomus leopardus* (Lacepède, 1802)	Japan	Ogawa et al. (2021) [2]
Benedenia epinepheli (Yamaguti, 1937) Meserve, 1938	EU707803	*Epinephelus* sp.	Vietnam	NA
Benedenia sciaenae (Van Beneden, 1852) Odhner, 1905	FJ971970	*Argyrosomus japonicus* (Temminck and Schlegel, 1843)	Australia	Perkins et al. (2009) [3]
Benedenia sekii (Yamaguti, 1937) Meserve, 1938	FJ971971	*Pagrus auratus* (Forster, 1801)	Australia	Perkins et al. (2009) [3]
Benedenia seriolae (Yamaguti, 1934) Meserve, 1938	AY033941	*Seriola quinqueradiata* Temminck and Schlegel, 1845	Japan	Whittington et al. (2001) [4]
Benedeniella posterocolpa (Hargis, 1955) Yamaguti, 1963	FJ971975	*Rhinoptera bonasus* (Mitchill, 1815)	USA	Perkins et al. (2009) [3]
Capsala laevis (Verrill, 1875) Johnston, 1929	JN980396	*Istiophorus platypterus* (Shaw, 1792)	China	NA
Capsala martinieri Bosc, 1811	AF382053	*Mola mola* (Linnaeus, 1758)	UK	Olson and Littlewood (2002) [5]
Capsala pricei Hidalgo-Escalente, 1958	JN980397	*Istiophorus platypterus* (Shaw, 1792)	China	NA
Capsaloides magnaspinosus Price, 1939	FJ971984	*Kajikia audax* (Philippi, 1887)	Australia	Perkins et al. (2009) [3]
Dioncopseudobenedenia elongata Nitta, 2020	LC536938	*Naso unicornis* (Forsskål, 1775)	Japan	Nitta (2020) [6]
Dioncopseudobenedenia kala Yamaguti, 1965	FJ971989	*Naso unicornis* (Forsskål, 1775)	New Caledonia	Perkins et al. (2009) [3]
Encotyllabe caballeroi Velasquez, 1977	AF026112	*Gymnocranius audleyi* Ogilby, 1916	Australia	Mollaret et al. (1997) [7]
Encotyllabe chironemi Robinson, 1961	AF382054	*Chironemus marmoratus* Günther, 1860	Australia	Olson and Littlewood (2002) [5]

Entobdella hippoglossi (Müller, 1776) Blainville, 1818	AF382055	*Hippoglossus hippoglossus* (Linnaeus, 1758)	UK	Olson and Littlewood (2002) [5]
Entobdella soleae (Van Beneden and Hesse, 1863)	AY486152	*Solea solea* (Linnaeus, 1758)	UK	Whittington et al. (2004) [8]
Entobdella stenolepis Kearn, Whittington and Evans-Gowing, 2007	FJ971991	*Hippoglossus stenolepis* Schmidt, 1904	Canada	Perkins et al. (2009) [3]
Gracilobenedenia hichi Nitta, 2021	LC602799	*Heteropriacanthus carolinus* (Cuvier, 1829)	Japan	Nitta (2021) [9]
Gracilobenedenia kuremibai Nitta, 2021	LC602797	*Plectorhinchus chaetodonoides* Lacépède, 1801	Japan	Nitta (2021) [9]
Gracilobenedenia lutjani (Whittington and Kearn, 1993) Nitta, 2021	LC602796	*Lutjanus fulviflamma* (Forsskål, 1775)	Japan	Nitta (2021) [9]
Gracilobenedenia rohdei (Whittington, Kearn and Beverley-Burton, 1994) Nitta, 2021	AY033940	*Lutjanus carponotatus* (Richardson, 1842)	Australia	Whittington et al. (2001) [4]
Interniloculus chilensis Interniloculus chilensis Suriano and Beverley-Burton, 1979	FJ971997	*Sebastes capensis* (Gmelin, 1789)	Chile	Perkins et al. (2009) [3]
Listrocephalos corona (Hargis, 1955) Bullard, Payne and Braswell, 2004	FJ971998	*Hypanus sabinus* (Lesueur, 1824)	USA	Perkins et al. (2009) [3]
Listrocephalos kearni Bullard, Payne and Braswell, 2004	FJ971999	*Dasyatis brevis* (Garman, 1880)	Mexico	Perkins et al. (2009) [3]
Macrophyllida sp.	FJ972000	*Hemigaleus microstoma* Bleeker, 1852	Australia	Perkins et al. (2009) [3]
Mediavagina sp.	FJ972019	*Nemadactylus valenciennesi* (Whitley, 1937)	Australia	Perkins et al. (2009) [3]
Megalobenedenia helicoleni (Woolcock, 1936)	FJ972001	*Helicolenus percoides* (Richardson and Solander, 1842)	Australia	Perkins et al. (2009) [3]
Metabenedeniella parva Horton and Whittington, 1994	LC602800	*Plectorhinchus chaetodonoides* Lacépède, 1801	Japan	Nitta (2021) [9]
Nasicola klawei (Stunkard, 1962) Yamaguti, 1968	HQ721184	*Thunnus albacares* (Bonnaterre, 1788)	USA	Bullard et al. (2011) [10]
Neobenedenia girellae (Hargis, 1955) Yamaguti, 1963	MG193664	*Neocirrhites armatus* (Castelnau, 1873)	Australia	Brazenor et al. (2018) [11]

Neobenedenia melleni (MacCallum, 1927) Yamaguti, 1963	FJ972005	*Sphoeroides annulatus* (Jenyns, 1842)	Mexico	Perkins et al. (2009) [3]
Neoentobdella australis (Kearn, 1978)	AF026108	*Taeniura lymna* (Forsskål, 1775)	Australia	Mollaret et al. (1997) [7]
Neoentobdella natans Kearn and Whittington, 2005	FJ972009	*Pastinachus sephen* (Forsskål, 1775)	Australia	Perkins et al. (2009) [3]
Neoentobdella taiwanensis Whittington and Kearn, 2009	FJ972010	*Taeniurops meyeni* Müller and Henle, 1841	Taiwan	Perkins et al. (2009) [3]
Nitzschia sturionis (Abildgaard, 1794) Krøyer, 1852	FJ972011	*Huso huso* (Linnaeus, 1758)	Russia	Perkins et al. (2009) [3]
Pseudonitzschia uku Yamaguti, 1965	FJ972013	*Aprion virescens* Valenciennes, 1830	New Caledonia	Perkins et al. (2009) [3]
***S. lamothei* Pérez-Ponce de Léon and Mendoza-Garfias, 2000**	**PZ323883**	***Parapsettus panamensis* (Steindachner, 1876)**	**Peru**	**Present study**
***S. micrancyra* Cezar, Luque and Amato, 1999**	**PZ328782**	***Chaetodipterus faber* (Broussonet, 1782)**	**Brazil**	**Present study**
Tareenia acanthopagri Hussey, 1986	FJ971967	*Sparidentex hasta* (Valenciennes, 1830)	Kuwait	Perkins et al. (2009) [3]
Tristoma integrum Diesing, 1850	OQ349751	*Xiphias gladius* Linnaeus, 1758	Algeria	Gastineau et al. (2023) [12]
Haplocotyle japonica Nitta and Nagasawa, 2017	LC150819*	*Rhinobatos hynnicephalus* Richardson, 1846	Japan	Nitta and Nagasawa (2017) [13]
Heterocotyle capricornensis Chisholm and Whittington, 1996	AF348360*	*Himantura fai* Jordan and Seale, 1906	Australia	Chisholm et al. (2001) [14]

Supplementary Table 2. Isolate sequences used in cox1 molecular analyses associated with their GenBank accession numbers, host, locality and reference. * Indicates sequence used as outgroup. NA: not available.

Species	GenBank Access Number	Host	Locality	Reference
Armatobenedenia armata Armatobenedenia armata (Nitta, 2019) Nitta, 2021	LC408960	*Lethrinus haematopterus* Temminck and Schlegel, 1844	Japan	Nitta (2019) [15]
Benedenia hoshinai Ogawa, 1984	EF055880	*Oplegnathus fasciatus* (Temminck and Schlegel, 1844)	NA	Kang et al. (2012) [16]
Benedenia humboldti Baeza, Sepúlveda and González, 2019	MK599467	*Seriola lalandi* Valenciennes, 1833	Chile	Baeza et al. (2019) [17]
Benedenia seriolae (Yamaguti, 1934) Meserve, 1938	AP019641	*Seriola* sp.	Japan	Kawato et al. (2019) [18]
Capsala pricei Hidalgo-Escalente, 1958	NC047185	NA	NA	NA
Dioncopseudobenedenia elongata Nitta, 2020	LC536943	*Naso unicornis* (Forsskål, 1775)	Japan	Nitta (2020) [6]
Dioncopseudobenedenia kala Yamaguti, 1965	LC540650	*Naso unicornis* (Forsskål, 1775)	Japan	Nitta (2020) [6]
Encotyllabe percussa Morey, Viana, Chota and Chero, 2024	PP919045	*Lethrinus nebulosus* (Forsskål, 1775)	Oman	Morales-Ávila et al. (2024) [19]
Encotyllabe vallei Monticelli, 1907	OR148271	*Sparus aurata* Linnaeus, 1758	Algeria	Zedam et al. (2023) [20]
Gracilobenedenia hichi Nitta, 2021	LC602811	*Priacanthus hamrur* (Fabricius, 1775)	Japan	Nitta (2021) [9]
Gracilobenedenia kuremibai Nitta, 2021	LC602810	*Plectorhinchus chaetodonoides* Lacepède, 1801	Japan	Nitta (2021) [9]
Gracilobenedenia lutjani (Whittington and Kearn, 1993) Nitta, 2021	LC602809	*Lutjanus vitta* (Quoy and Gaimard, 1824)	Japan	Nitta (2021) [9]
Metalobenedenia parva Horton and Whittington, 1994	LC602812	*Plectorhinchus chaetodonoides* Lacepède, 1801	Japan	Nitta (2021) [9]
Neobenedenia melleni (MacCallum, 1927) Yamaguti, 1963	JQ038228	*Seriola dumerili* (Risso, 1810)	China	Zhang et al. (2014) [21]
***S. lamothei* Pérez-Ponce de Léon and Mendoza-Garfias, 2000**	**PZ284680**	***Parapsettus panamensis* (Steindachner, 1876)**	**Peru**	**Present study**

***S. micrancyra* Cezar, Luque and Amato, 1999**	**PZ284734**	***Chaetodipterus faber* (Broussonet, 1782)**	**Brazil**	**Present study**
Neoheterocotyle quadrispinata Nitta, 2019*	LC469716	*Rhinobatos hynnicephalus* Richardson, 1846	Japan	Nitta (2019) [22]

References

1. Nitta M (2019) A new species of *Benedenia* Diesing, 1858 (Monogenea: Capsalidae) parasitic on *Lethrinus haematopterus* Temminck and Schlegel (Perciformes: Lethrinidae) from Japan. Syst Parasitol 96: 199–205. https://doi.org/10.1007/s11230-019-09840-4

2. Ogawa K, Mizuochi H, Yamaguchi T, Shirakashi S, Asai N, Agawa Y (2021) *Benedenia akajin* n. sp. (Monogenea: Capsalidae) from leopard coral grouper *Plectropomus leopardus* reared in Okinawa Prefecture, Japan. Fish Pathol 55: 117–124. https://doi.org/10.3147/jsfp.55.117

3. Perkins EM, Donnellan SC, Bertozzi T, Chisholm LA, Whittington ID (2009) Looks can deceive: molecular phylogeny of a family of flatworm ectoparasites (Monogenea: Capsalidae) does not reflect current morphological classification. Mol Phylogenet Evol 52(3): 705–714. https://doi.org/10.1016/j.ympev.2009.05.008

4. Whittington ID, Corneillie S, Talbot C, Morgan JAT, Adlard RD (2001) Infections of *Seriola quinqueradiata* Temminck and Schlegel and *S. dumerili* (Risso) in Japan by *Benedenia seriolae* (Monogenea) confirmed by morphology and 28S ribosomal DNA analysis. J Fish Dis 24: 421–425. https://doi.org/10.1046/j.1365-2761.2001.00309.x

5. Olson PD, Littlewood DT (2002) Phylogenetics of the Monogenea - evidence from a medley of molecules. Int J Parasitol 32(3): 233–244. https://doi.org/10.1016/s0020-7519(01)00328-9
6. Nitta M (2020) Two species of *Dioncopseudobenedenia* Yamaguti, 1965 (Monogenea: Capsalidae) from *Naso unicornis* (Forsskål) (Acanthuridae) and *Coryphaena hippurus* Linnaeus (Coryphaenidae) in western Japan, with a description of *D. elongata* n. sp. from *N. unicornis*. Syst Parasitol 97: 681–690. https://doi.org/10.1007/s11230-020-09941-5
7. Mollaret I, Jamieson BG, Adlard RD, Hugall A, Lecointre G, Chombard C, Justine JL (1997) Phylogenetic analysis of the Monogenea and their relationships with Digenea and Eucestoda inferred from 28S rDNA sequences. Mol Biochem Parasitol 90: 433–438. https://doi.org/10.1016/S0166-6851(97)00176-X
8. Whittington ID, Deveney MR, Morgan JA, Chisholm LA, Adlard RD (2004) A preliminary phylogenetic analysis of the Capsalidae (Platyhelminthes: Monogenea: Monopisthocotylea) inferred from large subunit rDNA sequences. Parasitol 128(5): 511–519. https://doi.org/10.1017/s0031182004004901
9. Nitta M (2021) Capsalids (Platyhelminthes: Monogenea) from marine fishes off Okinawa in Japan with the proposal of two new genera. Parasitol Int 85: 102448. https://doi.org/10.1016/j.parint.2021.102448
10. Bullard SA, Olivares-Fuster O, Benz GW, Arias CR (2011) Molecules infer origins of ectoparasite infrapopulations on tuna. Parasitol Int 60(4): 447–451. https://doi.org/10.1016/j.parint.2011.07.016

11. Brazenor AK, Saunders RJ, Miller TL, Hutson KS (2018). Morphological variation in the cosmopolitan fish parasite *Neobenedenia girellae* (Capsalidae: Monogenea). Int J Parasitol 48(2): 125–134. https://doi.org/10.1016/j.ijpara.2017.07.009

12. Gastineau R, Bouguerche C, Tazerouti F, Justine JL (2023) Morphological and molecular characterisation of *Tristoma integrum* Diesing, 1850 (Monogenea: Capsalidae), including its complete mitogenome. Parasite 30(16). https://doi.org/10.1051/parasite/2023016

13. Nitta M, Nagasawa K (2017) *Haplocotyle japonica* n. gen., n. sp. (Monogenea: Microbothriidae) parasitic on *Rhinobatos hynnicephalus* (Elasmobranchii: Rajiformes: Rhinobatidae) in Japanese waters. Spec Divers 22: 117-125. https://doi.org/10.12782/specdiv.22.117

14. Chisholm LA, Morgan JA, Adlard RD, Whittington ID (2001) Phylogenetic analysis of the Monocotylidae (Monogenea) inferred from 28S rDNA sequences. Int J Parasitol 31(13): 1537–1547. https://doi.org/10.1016/s0020-7519(01)00313-7

15. Nitta M (2019) A new species of *Benedenia* Diesing, 1858 (Monogenea: Capsalidae) parasitic on *Lethrinus haematopterus* Temminck and Schlegel (Perciformes: Lethrinidae) from Japan. Syst Parasitol 96: 199–205. https://doi.org/10.1007/s11230-019-09840-4

16. Kang S, Kim J, Lee J, Kim S, Min GS, Park JK (2012) The complete mitochondrial genome of an ectoparasitic monopisthocotylean fluke *Benedenia hoshinai* (Monogenea: Platyhelminthes). Mitochondrial DNA 23(3): 176–178. https://doi.org/10.3109/19401736.2012.668900

17. Baeza JA, Sepúlveda FA, González MT (2019) The complete mitochondrial genome and description of a new cryptic species of *Benedenia* Diesing, 1858 (Monogenea: Capsalidae), a major pathogen infecting the yellowtail kingfish *Seriola lalandi* Valenciennes in the South-East Pacific. Parasit Vectors 12: 490. https://doi.org/10.1186/s13071-019-3711-5

18. Kawato S, Kobayashi K, Shirakashi S, Yanagi S, Fukuda Y, Yamashita H, Nozaki R, Hirono I, Kondo H (2019) Phylogenetic Analysis with Complete Mitochondrial Genome Sequences of *Benedenia seriolae* Specimens Derived from Japanese Seriola spp.. Fish Pathol 54: 27–33. https://doi.org/10.3147/jsfp.54.27

19. Morales-Ávila JR, Al Jufaili S, Ogawa K (2024) Morpho-molecular characterization and phylogenetic relationships of *Encotyllabe percussa* n. sp. (Monogenea: Capsalidae) from the spangled emperor *Lethrinus nebulosus* (Teleostei: Lethrinidae). Syst Parasitol 101: 69. https://doi.org/10.1007/s11230-024-10193-w

20. Zedam FZ, Bouguerche C, Ahmed M, Tazerouti F (2023) Morphological and molecular characterization of *Encotyllabe vallei* Monticelli, 1907 (Monopisthocotylea, Monogenea) from the gilthead seabream *Sparus aurata* Linnaeus (Teleostei, Sparidae) from the southwestern Mediterranean and notes on host specificity of the genus *Encotyllabe* Diesing, 1850. J Helminthol 97: e82. https://doi.org/10.1017/S0022149X23000688

21. Zhang J, Wu X, Li Y, Zhao M, Xie M, Li A (2014). The complete mitochondrial genome of *Neobenedenia melleni* (Platyhelminthes: Monogenea): mitochondrial gene content, arrangement and composition compared with two *Benedenia* species. Mol Biol Rep 41(10): 6583–6589. https://doi.org/10.1007/s11033-014-3542-6

22. Nitta M (2019) A New Monocotylid Species, *Neoheterocotyle quadrispinata* n. sp. (Monogenea), Infecting Gills of *Rhinobatos hynnicephalus* in Japan. Spec Divers 24: 145–150. https://doi.org/10.12782/specdiv.24.145
